# The Effect of β-Hydroxy-β-Methyl Butyrate (HMB) upon Acute Fed-State Muscle Protein Turnover in Older Men and Women: A Randomized Double-Blind Controlled Crossover Clinical Trial

**DOI:** 10.3390/nu18091449

**Published:** 2026-04-30

**Authors:** Kenneth Smith, Haitham Abdullah, Supreeth Rudrappa, Amanda Gates, Jonathan Lewis, Iskandar Idris, Joseph J. Bass, Hannah Crossland, Daniel J. Wilkinson, Min Tian, Deborah S. Hustead, Geraldine E. Baggs, Suzette L. Pereira, Bethan E. Phillips, Philip J. Atherton

**Affiliations:** 1 MRC-Versus Arthritis Centre for Musculoskeletal Ageing Research and Nottingham NIHR BRC, School of Medicine, University of Nottingham, Derby DE22 3DT, UK; ken.smith@nottingham.ac.uk (K.S.); haithamabdulla@gmail.com (H.A.); supreeth.rudrappa@nhs.net (S.R.); amanda.gates@nottingham.ac.uk (A.G.); iskandar.idris@nottingham.ac.uk (I.I.); hannah.crossland2@nottingham.ac.uk (H.C.); daniel.wilkinson@nottingham.ac.uk (D.J.W.); beth.phillips@nottingham.ac.uk (B.E.P.); 2 Department of Diabetes and Endocrinology, Royal Derby Hospital, Derby DE22 3NE, UK; 3 28 Cabot Ct., Wayne, PA 19087, USA; min.tian26@gmail.com; 4 7575 Lilyhill Ct., Worthington, OH 43085, USA; dhustead@insight.rr.com; 5 Abbott Nutrition, Research & Development, Columbus, OH 43219, USA; geraldine.baggs@abbott.com (G.E.B.); suzette.pereira@abbott.com (S.L.P.)

**Keywords:** skeletal muscle, beta-hydroxy-beta-methylbutyrate (HMB), muscle protein synthesis (MPS), muscle protein breakdown (MPB), anabolic resistance, aging

## Abstract

**Background/Objectives**: Anabolic resistance is thought to underlie muscle loss in sarcopenia. Here, we investigated the adjuvant role of beta-hydroxy-beta-methylbutyrate (HMB), a leucine metabolite, on the acute muscle anabolic response to oral protein supplementation in older adults. **Methods**: A total of 24 community-dwelling older adults (68.5 ± 0.6 years; 13 men, 11 women) were randomized in a cross-over double-blind design to 40 g whey protein (Control) or 40 g whey protein with 3 g calcium–HMB (HMB). Subjects received a primed constant infusion of ^13^C_6_ phenylalanine to assess muscle protein synthesis (MPS, by tracer incorporation in myofibrils) and muscle protein breakdown (MPB, via arterio-venous dilution) at baseline and post supplementation. Fasted and 3 h fed-state plasma HMB, aminoacidemia, rates of MPS, MPB, limb and muscle blood flow were measured. **Results**: In all subjects, both interventions displayed significant increases in MPS in response to feeding [fasted to 3 h-fed change (mean ± SEM, standard error of the mean). Males: control, +0.032 ± 0.006%.h^−1^; HMB, +0.023 ± 0.004%.h^−1^; females: control, +0.023 ± 0.006%.h^−1^; HMB, +0.038 ± 0.006%.h^−1^, *p* < 0.05]. In older women, the addition of HMB further enhanced the MPS response (fasted to 3 h-fed change, *p* = 0.0495) and area under the curve (*p* = 0.0364) versus protein alone. During the late-fed period, MPB significantly decreased in HMB versus control (*p* = 0.0298), and this was also observed when subjects were separated by sex (*p* = 0.0012). **Conclusions**: High-dose protein bolus feeding increased MPS in older adults. Surprisingly, 40 g whey did not maximize the anabolic response in older women, and HMB further increased the MPS feeding response to protein. HMB further suppressed the MPB feeding response over a longer period of time. Further work is needed to understand the apparent sexual dimorphic MPS response to high protein.

## 1. Introduction

Older age is associated with declines in skeletal muscle mass, strength and function, termed sarcopenia [1]. Involving motor unit remodelling and muscle fibre atrophy as a result of lifestyle, genetic and disease interactions, sarcopenia is a major hurdle to improving health-span against the backdrop of increasing lifespan. Reflecting this, recent years have seen a shift away from lifespan (a tangible end-point in tractable pre-clinical models) toward healthy years of life (health-span), compressing the period of the lifespan when frailty and disability increase substantially [2]. In being a tissue responsive to environmental cues (e.g., nutrition and loading) [3], muscle is an organ system that can be targeted to improve physical function in older age, as well as general health, reflecting relationships between low muscle mass/function, mortality and morbidity [4,5].

Intake of adequate dietary protein is crucial for the maintenance of muscle mass; it is most clearly exemplified in the circumstances of protein-deficient states [6,7]. It follows that consumption and appropriate uptake and utilization of dietary protein in skeletal muscle is pivotal to the maintenance of whole-body lean mass in the long-term. The acute responses in skeletal muscle to consumption of protein nutrition has been subject to intense study, yielding key mechanistic and kinetic aspects. It is now known that the intake of dietary protein rich in essential amino acids (EAAs) stimulates transient increases in muscle protein synthesis (MPS) by 2–3-fold [8,9], an effect waning ~2–3 h after eating [3,10]. The foundation of this anabolic response is to replenish amino acids (AA) redeployed from muscle for other metabolic purposes in-between meals e.g., gluconeogenesis [11]. Assuming that muscle AA repletion is equal to or in excess of depletion, muscle mass remains constant across much of life.

In older age, a phenomenon described as anabolic resistance disrupts this equilibrium, whereby a blunting of fed-state MPS is evident despite like-for-like feeding regimens in younger and older adults [12]. A regression analysis of studies showed that higher protein doses are needed to stimulate MPS in older men (~71 years) as compared to younger men (~22 years) [13]. While the underlying mechanisms leading to anabolic resistance remain ill-defined, impaired digestion/absorption [14], increased AA splanchnic uptake [15], muscle microvascular dysfunction [16], and myocellular cell signalling deficits [17] have all been reported. In addition, there appears to be sexual dimorphism in MPS response in older adults, with men demonstrating a higher postprandial response than women to the same feeding regimen [18,19]. This was partially attributed to a higher basal MPS rate in older women than men; however, signalling responses linked to MPS also differed between men and women [18]. A recent study also demonstrated a higher basal MPS rate in postmenopausal women as compared with premenopausal women, which contributes to lower overall MPS response to feeding [20].

Irrespective of the mechanisms, this has prompted research on optimising nutrition into older age through, for example, modifying protein dosing and quality [21], intake pattern [8], or nutritional adjuvants complementary to dietary protein intake [22]. One such nutritional adjuvant is beta-hydroxy-beta-methylbutyrate (HMB), a metabolite of the key signalling EAA leucine. HMB is clinically shown to preserve muscle mass and strength in older adults [23,24,25] and clinical populations [26]. These clinical benefits are attributed to the signalling properties of HMB on muscle metabolic pathways. HMB can stimulate MPS and attenuate muscle protein breakdown (MPB), even in the absence of EAA [27,28]. Mechanistically, HMB can activate MPS via the mTOR (mechanistic target of rapamycin) signaling pathway [27] and attenuate muscle wasting in cachexia models by downregulating the ubiquitin–proteasome degradation pathway [29,30]. Acknowledging this, we undertook a randomized double-blind controlled crossover trial to investigate the effects of HMB (~3 g) adjuvant to a large bolus (~40 g) of whey protein versus whey protein alone in cohorts of older men and women. The aim of this study was to determine if HMB adjuvant to a large bolus of dietary protein would augment skeletal muscle anabolic response, particularly the postprandial rate of MPS, in both male and female older adults.

## 2. Materials and Methods

### 2.1. Subjects

We aimed to recruit 14 healthy male and female volunteers (65–75 years of age, see Table 1 for subject demographics) from the local area via demographically targeted postal invites and advertisement in the local press. All volunteers underwent a comprehensive clinical examination and metabolic screening under the supervision of a trained medical doctor; this was conducted at the University of Nottingham Royal Derby Hospital Centre from April 2014 to December 2014. Subjects with metabolic disease, lower limb musculoskeletal abnormalities, acute cerebrovascular or cardiovascular disease, active malignancy, uncontrolled hypertension, BMI < 18 or >28 kg.m^−2^, on medications that impact muscle protein metabolism or modulate vascular tone or with known allergy to any of the study products were excluded. All volunteers were studied on two occasions at 21–28 days apart and were asked to refrain from any purposeful exercise for 3 days prior to the study. Volunteers were randomly assigned to ingest a supplement that contained 40 g whey protein alone (control) or in combination with 3 g calcium HMB (HMB) (TSI, Shanghai, China) (Appendix A) in two sequences: ‘control’ followed by ‘HMB’, or ‘HMB’ followed by ‘control’. They were blinded to the composition of the drink. Analysts were also blinded to the treatment. Products were packaged in clinically labelled sachets which looked identical.

Randomisation was performed following inclusion into the study in order of recruitment, from two folders of sealed envelopes, one folder for each sex. The study was approved by The University of Nottingham Faculty of Medicine and Health Science Research Ethics Committee (H12122013), conducted in line with the Declaration of Helsinki and registered as a https://clinicaltrials.gov/ (NCT02052232, approval date: 6 December 2013).

### 2.2. Conduct of the Study

#### 2.2.1. Reporting and Initial Preparation

On the morning of the first study day, volunteers reported to the Clinical Physiology Laboratory at 0800 h, following an overnight fast from 2200 h (water consumption ad libitum). Whole-body (i.e., total lean and fat mass) and regional measures of body composition (i.e., arm and leg lean muscle mass) were determined following a whole-body dual-energy X-ray absorptiometry (DXA) scan (GE Lunar Prodigy, GE Healthcare, Buckinghamshire, UK). Volunteers then lay semi-supine on a bed for approximately 6 h. Cannulae were inserted into the femoral artery and vein of one leg using ultrasound guidance (Philips iU22 Ultrasound, Bothell, WA, USA) for the collection of arterial and venous blood samples. A third cannula was inserted into the opposite forearm for the infusion of a stable isotope amino acid tracer for the measurement of muscle protein turnover, and a contrast reagent Sonovue^TM^ (Bracco, Courcouronnes, France) for the measurement of tissue perfusion via contrast-enhanced ultrasound (CEUS).

#### 2.2.2. Stable Isotopically Labelled Phenylalanine Infusion

**Figure 1:**
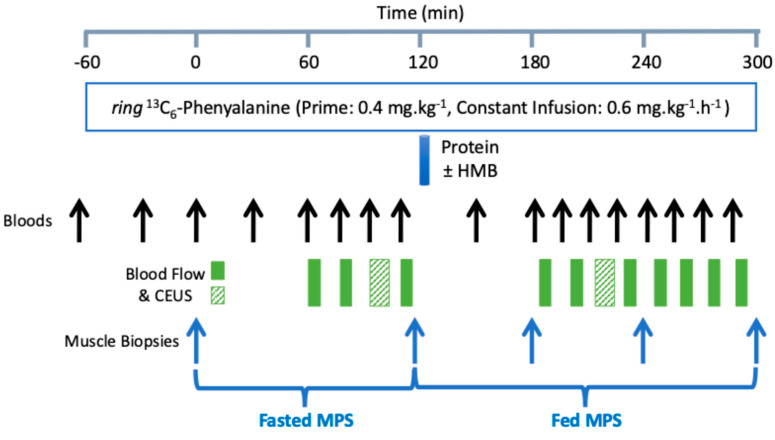
Schematic representation of the study day protocol.

Following collection of baseline venous and arterial blood samples, a primed (0.4 mg.kg^−1^) constant infusion (0.6 mg.kg^−1^.h^−1^) of ^13^C_6_ phenylalanine (Cambridge Isotope laboratories, Tewksbury, MA, USA) was initiated and continued for a total of 6 h (see Figure 1 for a schematic overview of the study protocol).

### 2.3. Feeding

During the first 3 h period, ‘baseline’ measurements were collected as indicated in Figure 1. The subjects then drank either the control or the HMB product. Each sachet of the protein supplement was dissolved in 8 oz water and provided to the subjects to be consumed in one bolus, as per the scheme in Figure 1. This enabled us to measure the postprandial effects of the feed over a 3 h window.

### 2.4. Blood Sampling and Leg Blood Flow

Blood samples were taken regularly throughout the study day for the measurement of plasma ^13^C_6_ phenylalanine and AA concentrations (see Figure 1 for timings). Over three periods, the artery and vein were sampled simultaneously at 60, 75, 90, 105 min (fasted); 180, 195, 210, 225 min (early-fed); and 240, 255, 270, 285 min (late-fed) for the assessment of leg phenylalanine kinetics. During these periods, three measures of leg (femoral artery) blood flow (LBF) were also taken using Doppler ultrasound and expressed per 100 g leg muscle mass. Regular venous samples were taken approximately every 30 min to assess the steady state of phenylalanine labelling. Blood samples were collected into Lithium Heparin and EDTA tubes, stored on ice, and then centrifuged. The plasma was aliquoted and stored at −80 °C in temperature-monitored alarmed freezers.

### 2.5. Muscle Biopsies

A muscle biopsy from the vastus lateralis was taken at 0 h (following the attainment of steady-state phenylalanine labelling) and at 2 h, immediately before feeding, to determine postabsorptive MPS. Three further muscle biopsies were taken hourly following provision of the oral nutrition to determine the impact of feeding, with and without HMB, on postprandial MPS. Muscle biopsies were obtained using our standard procedures using the conchotome technique [9,24]. Briefly, after infiltration of local anaesthetic, tissue was obtained using conchotome forceps before being frozen rapidly in cryogenic vials in liquid nitrogen with subsequent storage in a temperature-monitored −80 °C freezer.

### 2.6. Contrast-Enhanced Ultrasound (CEUS)

Approximately 90 min following initiation of the phenylalanine tracer infusion, a baseline postabsorptive measure of muscle microvascular blood volume (MBV) was conducted using CEUS (Philips iU22 Ultrasound, Bothell, WA, USA). In brief, Sonovue^TM^ (Bracco, Courcouronnes, France) contrast agent was infused via a peripheral vein at an initial rate of 2 mL.min^−1^ for 1 min followed by 1 mL.min^−1^ for a further 2 min. During this infusion, three contrast agent destruction–replenishment videos were recorded. Using Q-Lab software (Version 10.5, Phillips), the plateau of each replenishment curve was obtained, with the mean plateau (A-value) for each timepoint representing MBV [16]. A further measurement of MBV was made 90 min after the provisional of oral nutrition, when AA concentrations were maximal.

### 2.7. Cross-Over Details

At the end of the study, volunteers were monitored for 1 h and given a light meal before being transported home. Volunteers returned after 1 week for stitch removal and a check of all biopsy sites and then again ~3 weeks later to undertake the cross-over study on the contralateral leg (identical to the first and no DXA scan). A 4-week washout period was deemed more than adequate based on the known pharmacokinetics of HMB with a plasma half-life of 2.37 h, and it has been shown to reach baseline levels at ~9 h following ingestion [31].

### 2.8. Laboratory Analysis

#### 2.8.1. Determination of Plasma HMB, AA Concentrations, and Phenylalanine Enrichment

For all analysis, plasma was thawed and then centrifuged to remove fibrin clots. For the quantitation of AA concentrations, internal standards were added to samples before urease solution was added at room temperature for 20 min to remove urea interference. Samples were then de-proteinised using ice-cold ethanol before centrifugation at 13,000× *g* for 20 min at 4 °C. The supernatant containing plasma-free AA was decanted and evaporated at 90 °C under N_2_. Dried AAs were solubilised in 0.5 M HCl and the lipids extracted with ethyl acetate before being evaporated to dryness. AAs were derivatised by adding equal volumes of acetonitrile (ACN) and *N*-tert-Butyldimethylsilyl-*N*-methyltrifluoroacetamide (MTBSTFA) and heating at 90 °C for 60 min; they were subsequently cooled then transferred to autosampler vials for quantification against AA standard curves of a known concentration using gas chromatography–mass spectrometry (GC-MS). The enrichment [atom percent excess (APE)] of arterialized and venous ^13^C_6_ phenylalanine was determined using GC-MS, from the ratio of labelled to unlabelled phenylalanine (*m*/*z* 240 to 234) with reference to a standard curve of known enrichment (0–10 APE). Plasma HMB was analysed as previously described [27,28]. In brief, HMB was extracted from plasma with ethyl ether then back-extracted into 0.1 M phosphate buffer, dried, and analysed by GC-MS.

#### 2.8.2. Determination of Muscle and Albumin Protein-Bound Phenylalanine and Intracellular HMB and Free Phenylalanine Enrichment

Mixed muscle (~25 mg) was minced in 0.2 M perchloric acid using fine scissors, homogenised, and centrifuged to pellet the total protein. The supernatant was removed for determination of intramuscular Phe concentration and enrichment. The protein pellet was solubilised in 0.3 N NaOH for 20 min at 37 °C then transferred to a boiling tube, precipitated with perchloric acid, centrifuged, and washed twice with 70% ethanol before being hydrolysed overnight at 110 °C in a Dowex H^+^ resin, 0.1 M HCl slurry. The free amino acids were eluted from the resin in 2 M NH_4_OH, dried, and then derivatised as their *n*-acetyl, propyl esters (NAP). The L-[ring-^13^C_6_]-phenylalanine incorporation into myofibrillar protein was determined by gas chromatography–combustion isotope ratio mass spectrometry (GC–C-IRMS, Delta-plus XP, Thermo, Hemel Hampstead, UK). Separation was achieved on a 25 m · 0.25 mm · 1.0 μ-film DB 1701 capillary column (Agilent Technologies, West Lothian, UK). Gas chromatography–mass spectrometry (GC-MS, Agilent-5977a, Santa Clara, CA, USA) was used to determine muscle intracellular L-[ring-^13^C_6_]-phenylalanine enrichment. The supernatant containing the intramuscular free amino acid pool was precipitated, and the supernatant was purified by cation-exchange chromatography using Dowex H^+^ resin and derivatised as their tert-Butyldimethylsilyl (t-BDMS) derivatives before measurement of phenylalanine enrichment by GC-MS [9,28,29]. Plasma albumin was extracted as previously described; protein was precipitated into 10% trichloroacetic acid and the albumin protein back-extracted from the pellet into ethanol. The purified albumin was then hydrolysed and processed identically as for the mixed muscle pellet.

For intramuscular HMB, 20–30 mg of tissue was spiked with 20 µL of internal standard (D_6_-HMB; 10 µg/mL), and 125 µL of ultrapure water was added. The tissue was homogenised with a bead beater for 2 min and then centrifuged at 12,500 rpm for 2 min. Then, 375 µL of ice-cold methanol was added, and the sample was homogenised again before centrifuging for 10 min at 12,500 rpm. The supernatant was removed to a 2 mL autosampler vial and another 500 µL of methanol added to the muscle tissue pellet, vortexed, mixed, and centrifuged again at 12,500 rpm for 10 min. Supernatant was decanted and pooled with previous fraction. The pooled supernatant was then evaporated to dryness at 40 °C under nitrogen. Dried samples were resuspended in 100 µL of acetonitrile–ultrapure water (65:35) and analysed via LC-HRMS (Q Exactive, Thermo Scientific, Waltham, MA, USA). Concentration was determined from reference to a standard curve of a known concentration.

#### 2.8.3. Calculation of Fractional Synthesis Rates for Muscle and Albumin

The term fractional synthesis rate (FSR) of muscle is a measure of MPS and is used interchangeably with MPS. Mixed muscle protein FSR represents the fraction of labelled amino acid incorporated into the protein-bound pool over time. FSR was calculated via measurement of the increase in L-[ring-^13^C_6_]-phenylalanine enrichment into myofibrillar protein between two consecutive biopsies or plasma samples. The calculations used the standard equation:

FSR [expressed as %.h^−1^] = (ΔE_m_/E_p_ × 1/t) × 100,

where ΔE_m_ is the change in labelling of muscle-bound L-[ring-^13^C_6_]- phenylalanine between two biopsy samples, E_p_ is the mean L-[ring-^13^C_6_] free phenylalanine precursor enrichment in the intramuscular pool, and t is the time in hours between biopsies. A similar precursor-product approach was used for the calculation of albumin protein synthesis, using changes between bound Phe in plasma albumin over time relative to the average venous plasma ^13^C_6_ Phe, which was chosen to represent liver intracellular precursor labelling, over that period.

#### 2.8.4. Muscle Protein Breakdown Across the Leg

The rate of appearance (Ra) of phenylalanine (a surrogate measure of MPB calculated from the dilution of tracer across the leg) was calculated as follows:

(E_A_/E_V_ − 1) × LBF × C_A_,

with the rate of disappearance (i.e., MPS) calculated indirectly as the sum of MPB + (NB), where NB is (C_A_ − C_V_) × LBF; C_A_ and C_V_ are phenylalanine concentrations; and E_A_ and E_V_ are phenylalanine enrichments in the femoral artery and vein, respectively [27]. Phenylalanine kinetics were determined during the fasted/postabsorptive phase and then at two points during the fed/postprandial phase designated as early- and late-fed.

### 2.9. Statistical Analysis

The sample size was prospectively determined based on previous in-house studies detecting differences in muscle metabolism—MPS and MPB [27]. The primary outcome of the study was to understand the adjuvant effect of HMB on muscle metabolism as measured by MPS and MPB, with MPB being used for power analysis. For repeated measures of MPS in the same sample, the coefficient of variation (CV) is ~3.8%. For MPB, with a population CV of 15% and CV of laboratory techniques also of 15% (propagated error ~21%), we were able to detect (with 80% confidence at the 5% significance level) differences in rates of MPB after feeding of ±21% (i.e., 1 SD). Given these parameters, the smallest number of subjects needed to detect (with 80% confidence, 5% significance level) a one-way difference on a paired basis of 21% is 10. Secondary outcomes included micro- and macrovascular blood flow, and exploratory outcomes included plasma AA and HMB levels and albumin protein synthesis. The overall goal was to gather data that could be used in the design and outcome selection of future larger studies, including the choice of the population. Analysis was conducted using Prism 7 (GraphPad, San Diego, CA, USA). Data are presented as means ± SEM. Normality of distribution was tested using D’Agostino and Pearson Omnibus normality tests. Treatment effects overall and within gender were primary evaluations of interest, regardless of the presence of a treatment by gender interaction due to previous reports of sexual dimorphism [18,19]. Comparison between experiments was made via a repeated-measures ANOVA appropriate for crossover trials, with post-test analysis to determine significance. Within-treatment effects (e.g., fed vs. fasted state) were assessed using Student’s paired *t*-test. All hypothesis tests used α = 0.05. As there was one primary outcome, no adjustment for multiple testing of secondary or exploratory outcomes was employed.

## 3. Results

### 3.1. Subject Characteristics

**Figure 2:**
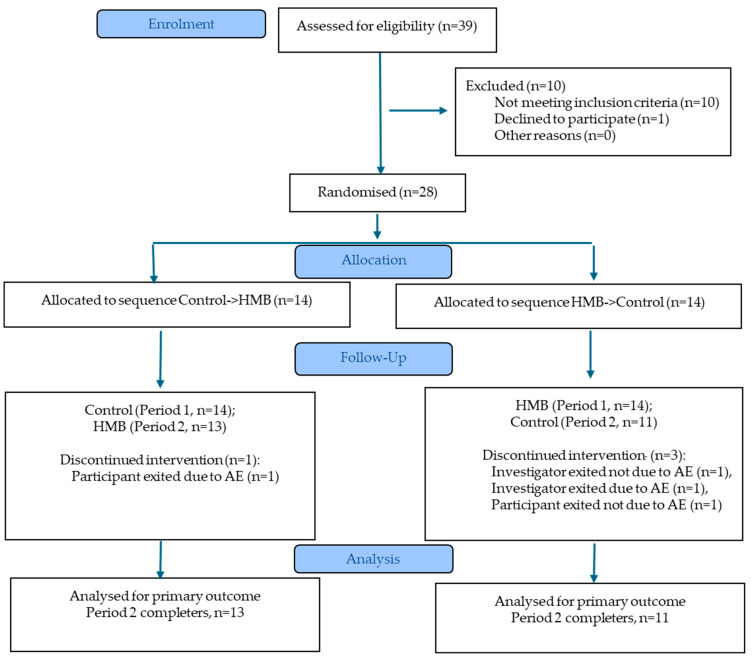
CONSORT flow diagram.

Fourteen subjects of each sex were recruited to the study, with 13 of 14 males and 11 of 14 females evaluable (Table 1, Figure 2). Four subjects withdrew from the study and did not complete the second visit. Data is presented only for subjects who completed both study periods. Consort check list indicated in Table S1.

### 3.2. Plasma and Intramuscular HMB

**Figure 3:**
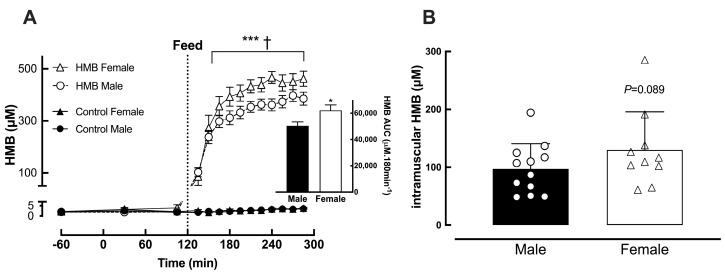
Plasma (**A**) and intramuscular (**B**) HMB concentrations pre and post feeding. Muscle biopsy was obtained 60 min post feeding. Values are presented as means ± SEM. Closed symbols represent the protein-only intervention (Control), and open symbols represent the protein + HMB intervention (HMB). Individual data from females are represented by triangles and circles represent data from males. HMB was significantly elevated in both plasma and intramuscular water (* *p* < 0.05) throughout the feeding period. HMB was not detected in intramuscular water from the protein-only intervention. *** *p* < 0.001 vs. control. † *p* < 0.001 vs. baseline for plasma HMB.

Plasma HMB concentrations rose rapidly following ingestion of the HMB supplement and remained elevated significantly (*p* < 0.001 vs. baseline) throughout the feeding period, plateauing over the last 60 min (Figure 3A). Plasma levels were numerically but not significantly higher in female subjects (Female: 417 ± 10 vs. Male: 386 ± 12 μM) (Figure 3A). This pattern was also observed for intramuscular HMB, being significantly elevated in the HMB treatment in both sexes (*p* < 0.001 vs. control) but numerically higher in females than in males 60 min after feeding (Female: 129 ± 66 vs. Male: 97 ± 44 μM, *p* = 0.089) (Figure 3B). When fed protein alone, plasma and intramuscular HMB concentrations remained the same as baseline throughout (~2–3 μM).

### 3.3. Plasma EAA Concentrations and Plasma/Intramuscular Phenylalanine Enrichment

**Figure 4:**
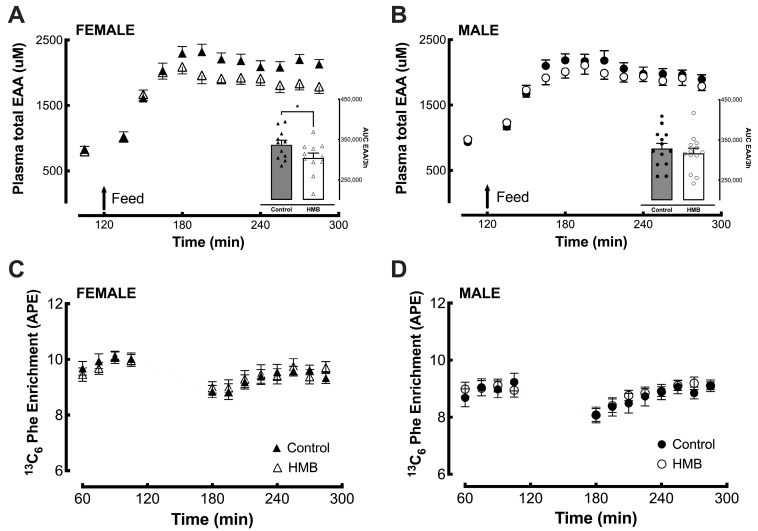
Arterial plasma total EAA concentrations and the area under the curve for EAAs post feed in female (**A**) and male (**B**) subjects; arterial plasma ^13^C_6_ Phe steady-state labelling throughout the time course of the fasted and fed periods in female (**C**) and male (**D**) subjects. Values are presented as means ± SEM. Closed symbols represent protein only (control), and open symbols represent protein + HMB (HMB). AUC above baseline and over the 3 h feeding period was numerically different only for females, * *p* = 0.076 HMB vs. control.

Plasma EAA concentrations were steady throughout the postabsorptive phase then rose rapidly in both interventions in response to the 40 g protein load. Concentrations plateaued approximately 45 min after feeding then fell slowly over the remaining postprandial period. The rate of appearance of EAA in the systemic circulation was similar in females (Figure 4A) and males (Figure 4B) and in both the control and HMB interventions; however, the peak in females fed HMB was significantly lower than when fed the control (*p* = 0.0096), although subsequent disposal of EAA followed a similar pattern of decay (Figure 4A). Calculation of AUC of EAA in the female subjects in the postprandial phase showed that this was numerically lower when given HMB versus control interventions (*p* = 0.076), and a similar effect was observed for branch chain amino acids (BCAAs) (*p* = 0.015). HMB did not affect the rise and fall of EAAs and BCAAs in males. The pattern of systemic non-essential amino acids (NEAAs) was not different with HMB in either gender.

Phenylalanine enrichment reached a steady state rapidly during the baseline period in all interventions and then fell slightly (~0.5 atoms percent excess, APE) following the feeds before recovering to baseline levels across the last two hours of the postprandial period in both feedings. Enrichment levels were marginally higher in females (~0.5–0.8 APE) but were identical in each gender between the control and HMB interventions (Figure 4C,D). Intramuscular phenylalanine was determined in all biopsies to provide an average precursor enrichment over the period between biopsies for the calculation of MPS. The mean enrichment was ~7 APE in the postabsorptive phase and rose slightly (by ~1–1.5 APE) over the postprandial period, with no difference between control and HMB studies. The enrichment was marginally higher in female muscle, likely reflecting the differences in circulating plasma phenylalanine enrichments.

### 3.4. Mixed Muscle Protein Synthesis (MPS)

**Figure 5:**
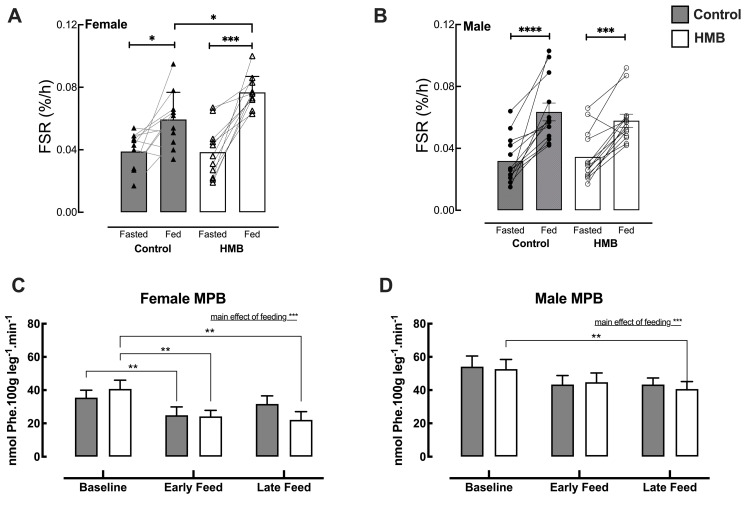
MPS as measured by mixed muscle fractional synthesis rate (FSR, %.h^−1^) in fasted and fed conditions in females (**A**) and males (**B**) with and without HMB. Muscle protein breakdown (MPB) in nmol Phe.100 g leg^−1^.min^−1^ under fasted and fed conditions in females (**C**) and males (**D**). Grey bars represent control, and white bars represent HMB. All fed FSR values were significantly different from fasted FSR, and in females, HMB-fed FSR was significantly higher than control-fed FSR. For MPB, early-fed relates to the period from 45 to 90 min post feeding, and late-fed relates to 135–180 min post feeding. Values are expressed as means ± SEM, and statistical analyses were carried out using a multiple-comparisons two-way ANOVA, with Sidak’s post hoc testing and significance set at *p* < 0.05, where * *p* < 0.05, ** *p* < 0.01 *** *p* < 0.001 **** *p* < 0.0001.

There were no significant differences in baseline MPS (as measured by FSR) by sex or treatment groups. When all subjects were combined, both treatments displayed a significant increase in MPS from its fasted value, with no significant treatment differences (Table 2). Similar results were obtained when treatments were separated by sex, showing significant increases in MPS in response to high-protein feeding (Table 2 and Figure 5A,B). In older men, there were no significant differences between treatments postprandially. However, in older women, the addition of HMB further increased the MPS response to protein versus protein alone (Figure 5A), with a significant increase in 0 h–3 h AUC (Table 2). Analysis of myofibrillar MPS followed the same pattern as that reported for mixed muscle MPS, with older women showing a significant postprandial increase in myofibrillar MPS when fed HMB versus control.

### 3.5. Muscle Protein Breakdown (MPB)

The rate of appearance (Ra) of phenylalanine, indicative of MPB, was numerically reduced during the postprandial phase in both sexes and treatments. When all subject data was combined, both treatments displayed a significant decrease in MPB from its fasted value during both the early-fed and late-fed periods. However, during the late-fed period, there was a significant difference between treatments, with HMB displaying lower MPB than control (*p* = 0.0298) (Table 3). In females, for early-fed periods, both treatments had a significant decrease in MPB from fasted values (Control, *p* = 0.0390; HMB: *p* = 0.0084). However, for the late-fed period, only the HMB intervention showed a significant decrease in MPB (*p* = 0.0012) (Table 3, Figure 5C). In the males, MPB was suppressed significantly for the late-fed period vs. fasted in response to the HMB intervention (*p* = 0.0037) but not the control (Table 3, Figure 5D).

### 3.6. Albumin Protein Synthesis

**Figure 6:**
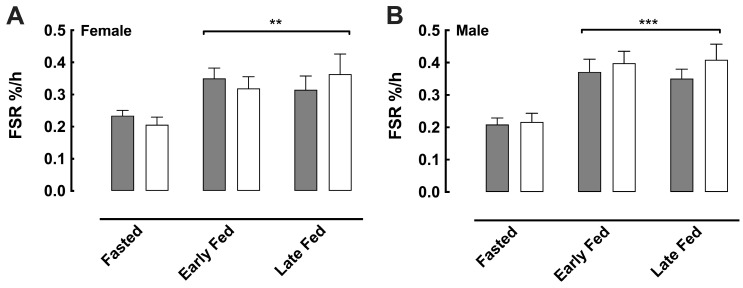
Albumin fractional synthesis rate (FSR, %.h^−1^) in fasted and fed conditions in females (**A**) and males (**B**). Grey bars represent the control and white bars HMB. Early-fed relates to the period from 45–90 min post feed, and late-fed relates to 135–180 min post feed. Values are expressed as means ± SEM. ** *p* < 0.01; *** *p* < 0.001.

Albumin protein synthesis in the fasted state was ~0.22%.h^−1^ across all sexes and treatments and increased significantly in response to feeding for both interventions over 1–2 h to 0.31–0.41%.h^−1^ and remained elevated over the final hour; however, there were no differences attributed to either sex or treatment (Figure 6).

### 3.7. Macro and Microvascular Blood Flow

Femoral artery (leg) blood flow (LBF) significantly increased during the early and late-fed postprandial phase in both treatments. When assessed by sex, this increase was significant in both treatments in females (control: baseline: 0.36 ± 0.09 to early-fed: 0.43 ± 0.05 and late-fed: 0.45 ± 0.11 L.min^−1^; HMB: 0.33 ± 0.06 to 0.42 ± 0.13 and 0.43 ± 0.09 L.min^−1^, all *p* < 0.05) but only in response to HMB treatment in males (control: 0.46 ± 0.14 to early-fed: 0.51 ± 0.07 and late-fed: 0.51 ± 0.09 L.min^−1^, *NS*; HMB: 0.47 ± 0.11 to early-fed: 0.55 ± 0.13 and late-fed: 0.54 ± 0.14 L.min^−1^, *p* < 0.05). Both postabsorptive and postprandial LBF values were significantly lower in females when compared to males. Conversely, microvascular blood volume (MBV), a surrogate for microvascular blood flow, was not changed in either treatment in response to the feed (control: fasted 0.63 ± 0.10 vs. fed 0.60 ± 0.08; HMB: fasted 0.60 ± 0.08 vs. fed 0.63 ± 0.07, Acoustic Index, *NS*), nor were there any significant differences between sexes.

### 3.8. Adverse Events

A total of five participants (two control, three HMB) experienced adverse events (AEs). One serious AE (acute coronary syndrome) was reported in the control group. Other AEs included skin injury, vomiting, and syncope. Most AEs were reported to be mild in severity except for syncope in a participant that was moderate in severity. These AEs were not directly related to product use.

## 4. Discussion

On the strength of data implicating both dietary protein and HMB as independent effectors of skeletal muscle anabolism, we investigated the impact of HMB as an adjuvant to a high-protein supplement on acute aspects of muscle protein metabolism. It is well known that there is a decline in anabolic response with age [12]. It has been proposed that to counter age-associated anabolic resistance, a fairly high bolus dose of protein (~0.4 g/kg bw/meal-equivalent to ~32 g protein/meal) is needed to maximally stimulate MPS in older adults [13,32]. However there is some evidence pointing to sexual dimorphism in MPS response to protein intake in women versus men across all age groups including older adults [18,19], middle-aged adults [33], and young exercising adults [34].

Thus, we reasoned that by employing a “maximally effective” protein dose (40 g whey protein bolus) in both older men and women, any additional effects beyond that of protein would be particularly important in developing interventions to address anabolic resistance. In brief, we report that HMB adjuvant to a 40 g whey protein bolus increased MPS beyond 40 g of protein alone in older women but not older men. The bolus dose of 40 g protein used equated to ~0.6 g protein/kg body weight (bw) for women and ~0.5 g protein/kg bw for men. Although it may appear that protein dosing tied to body weight could be a confounder for interpreting the MPS data, our statistical analysis model did include gender effects which accounted for the differences in protein intake and body composition driven by gender. These findings affirmprevious reports of sexual dimorphism in anabolic responses to protein intake in men and women [32] but also point to the potential potency of HMB as an adjuvant to enhance the anabolic response in older women.

Intake of protein leads to rises in plasma aminoacidemia, the profiles of which are often used to indicate protein bioavailability characteristics. Nonetheless, plasma AA profiles from protein intake should be interpreted with care due to (i) the influences of first pass splanchnic extraction (mainly consuming non-EAA), (ii) the influences of rates of peripheral tissue uptake of AA, and (iii) insulin-mediated suppression of whole-body protein breakdown reducing release of AA from cellular proteins [35]. Here, predominantly in older women, we observed differences between HMB and control feedings; while both treatments displayed similar increases in plasma EAA during the rise to peak, adjuvant HMB led to more rapid declines in plasma aminoacidemia. While it may seem intuitive that the greater area under the curve in EAA would indicate enhanced bio-availability, it could also indicate reduced systemic clearance [17]. As an example of this, when examining AA curves in younger and older adults following identical intake of AA, both peak similarly but tail off more slowly in older individuals, reflecting impaired tissue uptake/incorporation (or anabolic resistance) in old age [17]. The similar profile seen with adjuvant HMB may suggest more rapid uptake and utilization of AA in muscle/other tissues. Finally, that rapidly bioavailable plasma HMB was evident and to an apparent greater extent in females than males could reflect increased tissue uptake in males or could be reflective of sex-based differences in body size to plasma/HMB volumes per 3 gm HMB.

MPS is the primary endpoint reflecting protein accretion. Here, we observed robust increases in MPS in all subjects as well as in both sexes in all fed-state scenarios, a result to be expected given the substantial oral protein load provided even against the backdrop of anabolic resistance [13]. These data indicate, irrespective of all else, that larger doses (i.e., at the upper end of requirements for acute MPS responses [31]) of protein stimulate marked increases in MPS even in older age. Despite this, HMB was able to elicit effects even at a 40 g whey protein dose. Notably, adjuvant HMB augmented responses to 40 g protein feeding in older women. While the protein dose used in this study, particularly whey protein, may be higher than that typically encountered in habitual dietary patterns—especially among older adults with generally lower protein intakes—our results underscore the potential role of increasing protein intake in combination with HMB to promote muscle anabolism in older adults. Additionally, these findings may have implications for physically active older adults who consume higher protein as part of efforts to maintain muscle mass with advancing age.

Difference in MPS responses to protein nutrition in older men and women have been reported previously, with an earlier study reporting higher MPS response to protein in older men than women. The lower MPS response in women was attributed to higher rates of basal MPS in older women and differences in feeding-related anabolic signaling response (elements of the intracellular signaling pathways involved in regulation of MPS e.g., eIF4E and eIF4E-BP1) between older men and women [18,19]. We did not identify significant gender differences in basal MPS as previously reported [18]. With regard to HMB, the exact mechanisms tied to the augmented increase in the MPS in older women are unknown. Although hypothetical, it is possible that in women, HMB was able to activate protein synthesis via an mTOR-independent pathway via concurrent activation of phospholipase D2 [36], in addition to its well-known anabolic effect via the mTOR-dependent pathway [27]. HMB, in isolation, has been shown to stimulate MPS independent of leucine or protein, and this has been shown to occur in an insulin-independent manner [27]. Thus, anabolic effects beyond protein should not be unexpected. However, larger more adequately powered sex-stratified studies are needed to validate sex-specific findings identified in this study and understand the mechanism of action of HMB. 

We did not identify statistically significant differences in basal MPS between our older men and women, as previously reported [18]. Instead, here, enhanced fed-state MPS in females may represent a higher capacity for the stimulation of MPS (than in males) that could underlie enhanced capacity for muscle maintenance. In addition to muscle and other tissues responding with increases in protein synthesis, hepatic albumin synthesis [37] is a metabolic feature of the feeding response. Albumin deficiency is a biomarker of malnutrition, and its synthesis and maintenance of its vast pool size (~50% of plasma proteins) are important for the transportation of insoluble fatty acids, vitamins, and hormones and to maintain oncotic pressure. As expected, rates of plasma albumin protein synthesis were robustly responsive to feeding in our older subjects, irrespective of sex, while adjuvant HMB did not affect this response. As such, it may be that a ceiling effect on albumin protein synthesis was reached, or that HMB’s intrinsic or adjuvant effects are tissue-specific e.g., to skeletal muscle.

Alongside increasing MPS, HMB has also been shown to suppress MPB in humans [27,28,38] and in pre-clinical models of muscle catabolism. HMB can reduce MPB via multiple mechanistic avenues including the attenuation of proteasomal-mediated proteolysis, attenuated caspase levels, and inhibition of myonuclear apoptosis [29,30,39,40,41]. Here, we report that feeding protein alone could suppress MPB in all subjects only during the early-fed period (45–90 min post-feed), most likely due to the anti-proteolytic effect of insulin released in response to protein. In contrast, the addition of HMB extended the period over which MPB was suppressed, with suppression of MPBobserved during both the early- and late-fed (135–180 min) periods. This is in line with HMB’s previously described action in downregulating catabolic signaling pathways independent of insulin effect [29,30,39,40]. This effect of HMB on reducing MPB was also apparent when subjects were separated by sex. We previously showed that HMB-induced suppression of MPB is insulin-independent since HMB blunted MPB without inducing insulin secretion [27]. Thus, it can be speculated that this prolonged anti-catabolic effect observed with adjuvant HMB could further enhance protein accretion during postprandial conditions. Provision of HMB alone, or alongside lower doses of protein would help to further delineate such a contribution.

One limitation of our study is the evaluation of various outcomes without adjustment for multiple testing, increasing the risk of type I error (false positives). As adequate power was calculated only for the primary outcome, analyses of other outcomes for this study with a small sample size were evaluated to assess signals in the data. Another limitation is that the sample size may have been too low to adequately address sex-specific findings, due to the exploratory nature of this study. However, overall, the significant results show internal consistency. Confirmation of outcomes in bigger sex-stratified studies is recommended.

We demonstrate the effects of adjuvant HMB alongside a high dose of dietary protein in older individuals of both sexes. The reasons for elements of sexual dimorphic responses to adjuvant HMB (i.e., responses in protein turnover) require further interrogation. Nonetheless, data from this well-controlled cross-over trial indicate that even under what may be perceived as “saturating doses” of protein, HMB is able to elicit alterations in aspects of AA handling and metabolism, which are translated into favourable metabolic outcomes vis-à-vis muscle protein metabolism.

## 5. Conclusions

We conclude that high-dose whey protein bolus feeding increased MPS in older men and women. Surprisingly, whereas 40 g of whey protein was not able to overcome the anabolic resistance in older women, the addition of adjuvant HMB was able to further enhance the MPS feeding response to high protein. HMB also suppressed the MPB response to protein feeding over a longer period of time. These data provide mechanistic evidence for the clinical potential of HMB in conjunction with high protein to improve muscle anabolism in populations at greater risk of muscle loss and falls, especially older adults.

## Figures and Tables

**Table 1:** Characteristics of evaluable subjects.

Parameter	Male (*n* = 13)	Female (*n* = 11)
Age (years)	69.7 ± 2.8	67.2 ± 2.9
Height (m)	1.74 ± 0.05	1.64 ± 0.08
Weight (kg)	81.4 ± 5.7	67.7 ± 8.3
BMI (kg.m^−2^)	26.7 ± 1.8	25.1 ± 1.9
Total leg lean mass (kg)	18.3 ± 1.7	12.9 ± 1.9
Appendicular skeletal mass (ASM) (kg)	25.2 ± 2.1	16.9 ± 2.4
Skeletal muscle index (SMI) (kg.m^−2^)	8.3 ± 0.5	6.3 ± 0.6
Total lean mass (kg)	55.0 ± 3.2	38.8 ± 5.1
Total fat mass (kg)	23.3 ± 5.3	25.5 ± 5.8
SPPB score	10.8 ± 1.0	10.4 ± 1.1
Average grip strength (kg)	36.1 ± 10.6	23.7 ± 5.3

Values expressed as means ± SD. ASM was determined from the sum of lean tissue in the arms and legs, and SMI = ASM/height^2^.

**Table 2:** Muscle protein synthesis (measured by mixed muscle protein fractional synthesis rates).

	Baseline (0 h) FSR (%/h)	3 h Fed FSR (%/h)	FSR Change (0 h–3 h) (%/h)	AUC (0 h–3 h) (%)
All (*n* = 24)				
Control	0.035 ± 0.003	0.063 ± 0.004	0.028 ± 0.004 *	0.179 ± 0.010
HMB	0.036 ± 0.003	0.066 ± 0.003	0.030 ± 0.004 *	0.192 ± 0.009
Female (*n* = 11)				
Control	0.039 ± 0.004	0.062 ± 0.005	0.023 ± 0.006 *	0.176 ± 0.014
HMB	0.039 ± 0.005	0.077 ± 0.003	0.038 ± 0.006 * ^#^	0.206 ± 0.008 ^†^
Male (*n* = 13)				
Control	0.032 ± 0.004	0.064 ± 0.006	0.032 ± 0.006 *	0.182 ± 0.016
HMB	0.034 ± 0.004	0.058 ± 0.004	0.023 ± 0.004 *	0.181 ± 0.015

* Change from baseline, *p* < 0.050; ^#^ treatment comparison, *p* = 0.0495; ^†^ treatment comparison, *p* = 0.0364; Values expressed as mean ± SEM.

**Table 3:** Muscle protein breakdown (measured by the rate of appearance of phenylalanine).

	Fasted (nmol Phe/100 g leg/min)	Early-Fed (nmol Phe/100 g leg/min)	Late-Fed (nmol Phe/100 g leg/min)	Change (Early-Fed to Fasted)	Change (Late-Fed to Fasted)
All (*n* = 24)					
Control	45.6 ± 4.4	34.9 ± 4.1	38.0 ± 3.3	−10.7 ± 3.4 **	−7.6 ± 3.6 *
HMB	47.2 ± 4.1	35.3 ± 4.0	32.1 ± 3.8	−11.9 ± 3.5 **	−15.1 ± 2.7 ** ^†^
Female (*n* = 11)					
Control	35.5 ± 4.5	24.9 ± 5.0	31.6 ± 4.9	−10.6 ± 4.5 *	−3.8 ± 4.2
HMB	40.8 ± 5.3	24.2 ± 3.7	22.1 ± 5.0 *	−16.6 ± 5.1 **	−18.7 ± 4.2 **
Male (*n* = 13)					
Control	54.1 ± 6.4	43.3 ± 5.4	43.4 ± 3.9	−10.8 ± 5.2	−10.7 ± 5.7
HMB	52.6 ± 5.8	44.7 ± 5.6	40.6 ± 4.5	−7.9 ± 4.7	−12.0 ± 3.4 **

* *p* < 0.05, ** *p* < 0.01, paired *t*-test, ^†^ Treatment difference, *p* = 0.0298, Control: −7.14 ± 3.26; HMB: −15.48 ± 3.26 (*n* = 24) (repeated measures ANOVA), Values expressed as mean ± SEM.

## Data Availability

The original contributions presented in this study are included in the article. Further inquiries can be directed to the corresponding authors.

## Supplementary Materials

The following supporting information can be downloaded at: https://www.mdpi.com/article/10.3390/nu18091449/s1, Table S1: Consort checklist.

## Abbreviations

The following abbreviations are used in this manuscript:

HMB	Beta-hydroxy-beta-methylbutyrate
MPS	Muscle protein synthesis
MPB	Muscle protein breakdown
AA	Amino acids
EAA	Essential amino acids
Ra	Rate of appearance
FSR	Fractional synthesis rate
A-V	Arterial–venous
BMI	Body mass index
CEUS	Contrast-enhanced ultrasound
LBF	Leg blood flow
DXA	Dual-energy X-ray absorptiometry
APE	Atom percent excess
SPPB	Short physical performance battery
GS-MS	Gas chromatography–mass spectrometry

## Appendix A

**Table A1:** Product composition.

	Control	HMB
Calories	220	220
Whey protein concentrate (g)	40	40
Total carbohydrate	8	8
Sugar (g)	3	3
Dietary fibre (g)	<1	<1
Total fat (g)	3.5	3.5
Saturated fat (g)	1	1
Sodium (mg)	160	160
Potassium (mg)	320	320
Calcium–HMB (g)	0	3

1 sachet = 60 g powder.
